# Supplementary material for: Bidirectional relationship between olfaction and Parkinson’s disease
Source: medRxiv. 2023 Oct 19:2023.10.18.23297218. Preprint. [Version 1] doi: 10.1101/2023.10.18.23297218 (PMC10615003; doi:10.1101/2023.10.18.23297218)

Supplementary Figure 1 - Manhattan Plots of a) PD and b) ability to smell GWAS

a.

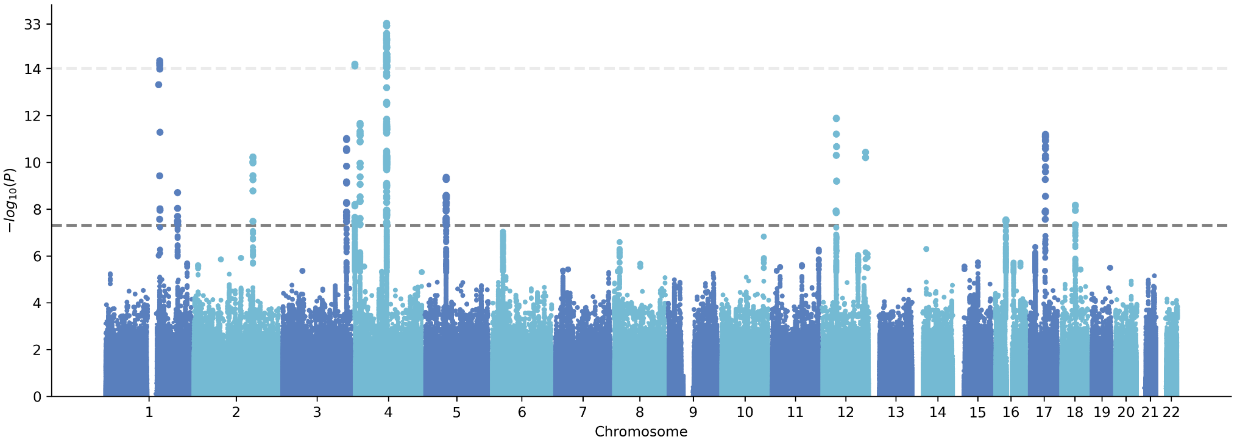

b.

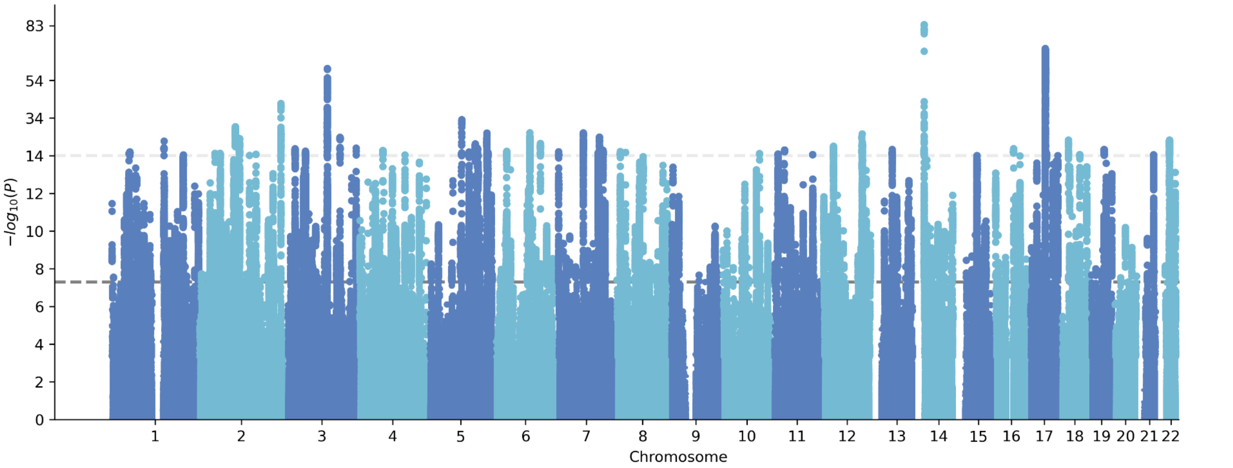

Supplementary Figure 2 - Forest plot of leave-one-out sensitivity results

PD on ability to smell

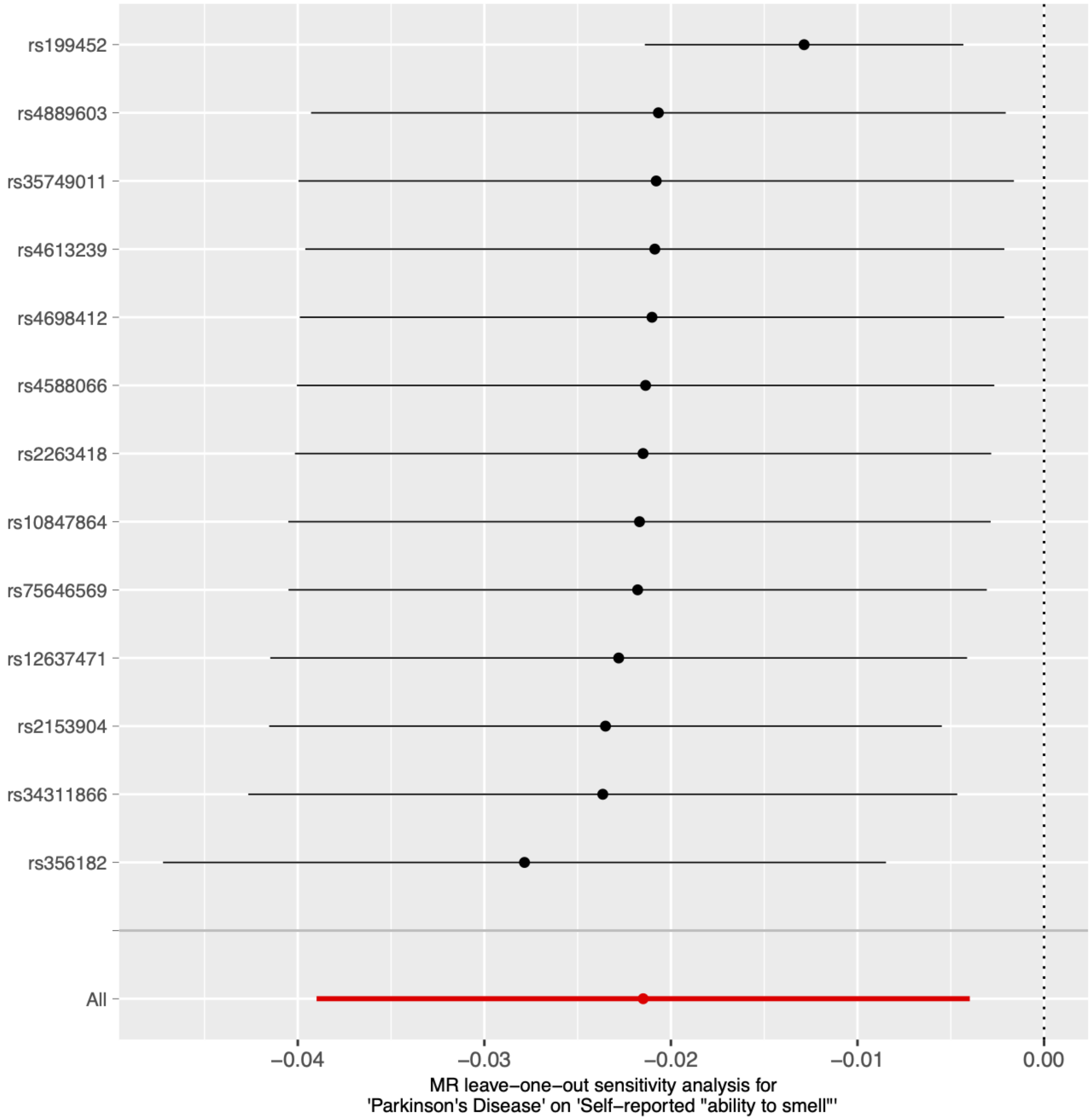

PD on ability to smell (outliers removed)

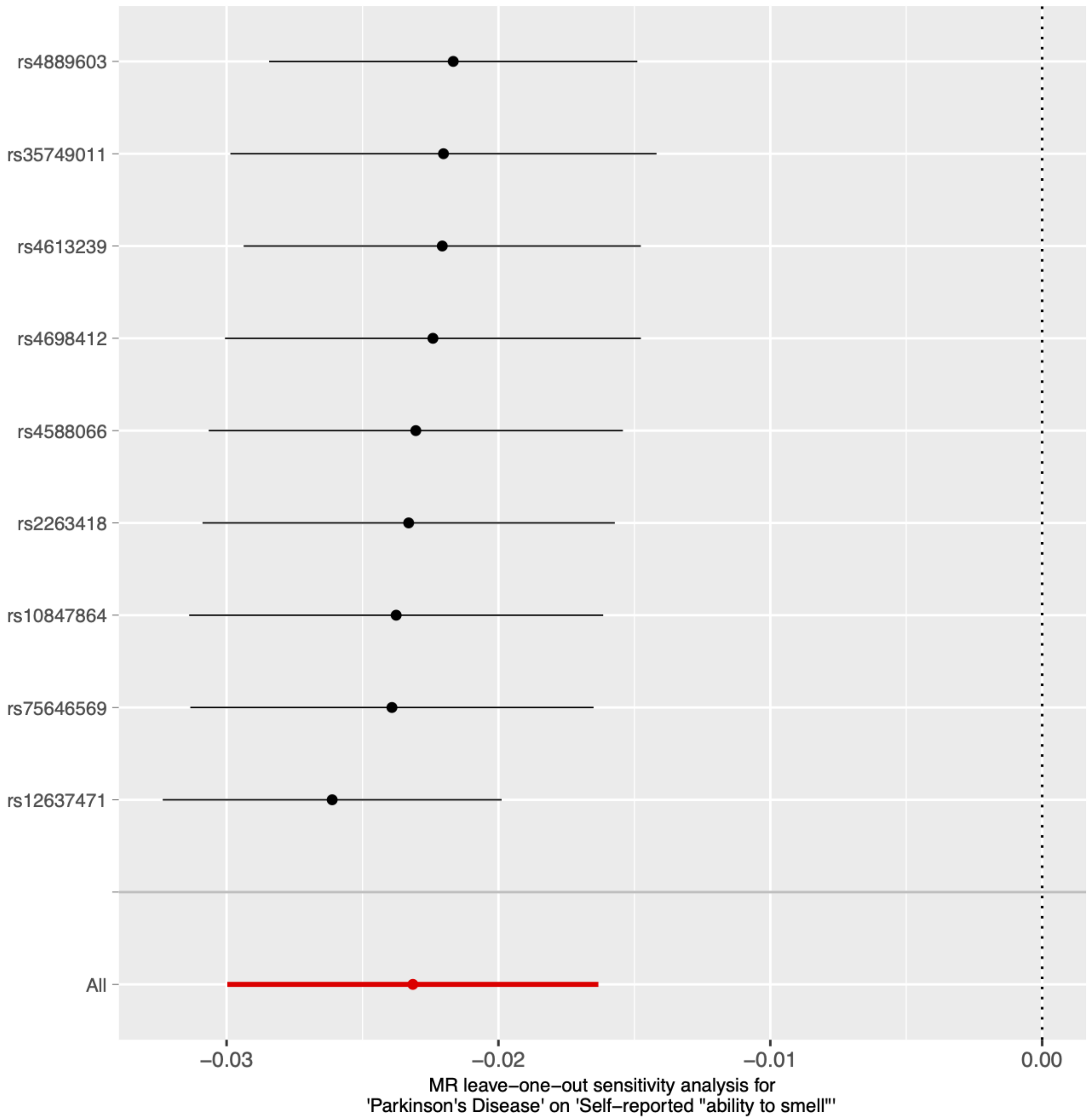

Ability to smell on PD

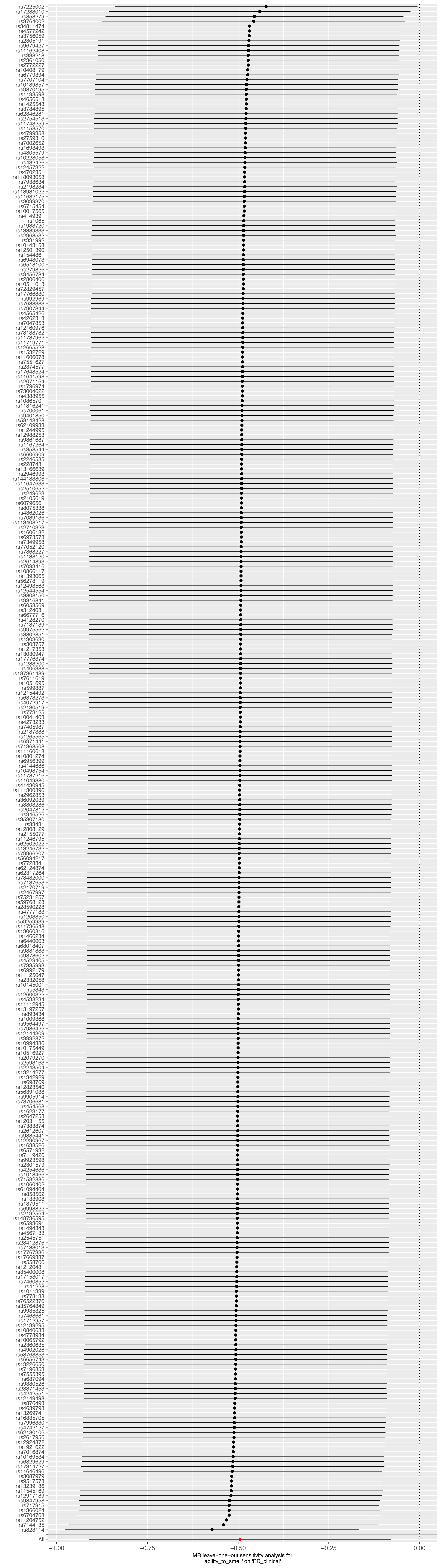
All
−1.00
−0.75
−0.50
−0.25
0.00
MR leave−one−out sensitivity analysis for
'ability_to_smell' on 'PD_clinical'

Ability to smell on PD (outliers removed)

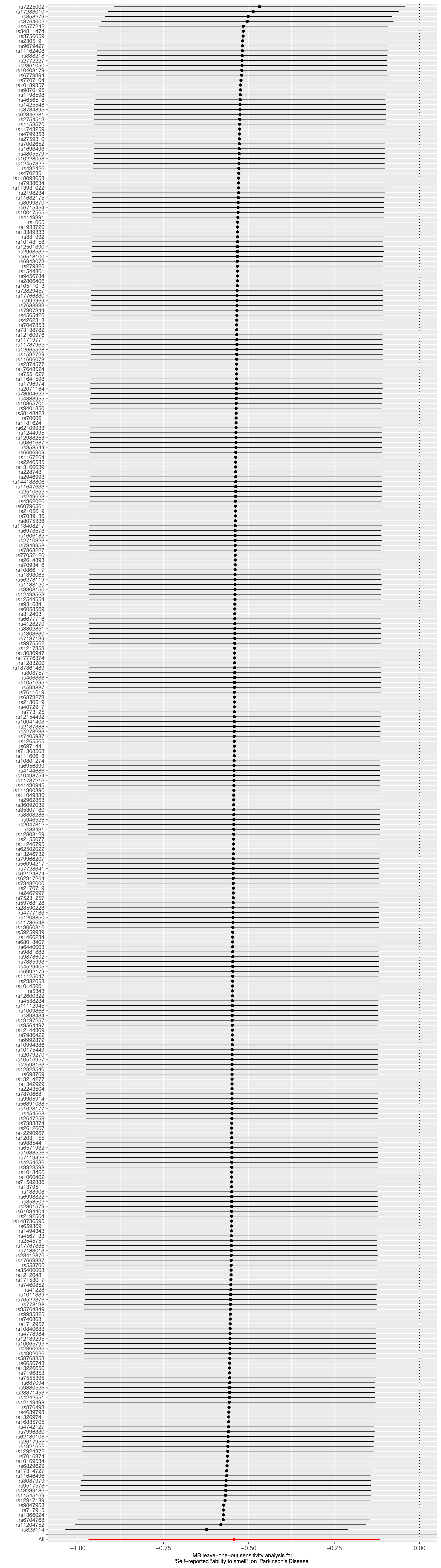

rs7225002
rs17283010
rs858279
rs3764002
rs4577242
rs34811474
rs3756059
rs2305191
rs9679427
rs11162408
rs338218
rs2772227
rs2361050
rs10408179
rs6779394
rs7707104
rs10189857
rs9870195
rs1198598
rs4656518
rs1425548
rs3784895
rs62346281
rs2754513
rs1158570
rs11743259
rs4799358
rs2759310
rs7002652
rs1693493
rs4805579
rs10228058
rs12457322
rs432426
rs4702351
rs118093058
rs7938634
rs113931022
rs2198234
rs11682175
rs3099370
rs6715454
rs10017565
rs4149391
rs1065
rs1933720
rs13389333
rs331992
rs10143158
rs12501390
rs2968532
rs6518100
rs6943073
rs279826
rs1544861
rs9456784
rs2806406
rs10511013
rs72829457
rs17766830
rs992969
rs7688383
rs7907344
rs4565426
rs4262318
rs7047853
rs73138782
rs12160976
rs11719771
rs11737962
rs12665528
rs1532729
rs11606078
rs2374577
rs17648524
rs7551627
rs11641598
rs1796974
rs2071164
rs73004622
rs4388955
rs10865701
rs9401850
rs58148428
rs700061
rs11816241
rs62109933
rs1244995
rs12988253
rs9861687
rs358544
rs6606909
rs1167264
rs2246585
rs13166639
rs2287431
rs2946993
rs144183806
rs11647633
rs2510652
rs249623
rs4362026
rs60796561
rs2105619
rs7039136
rs8075338
rs113408217
rs6973573
rs1606182
rs2710323
rs7349958
rs7868227
rs77052120
rs2614893
rs7093416
rs10866117
rs1393065
rs56278119
rs1138120
rs3808150
rs12493563
rs12544554
rs9316841
rs6058569
rs3124031
rs6677716
rs4128270
rs3802851
rs1303630
rs7137139
rs9975562
rs1217353
rs13030947
rs17776374
rs1283200
rs187361489
rs303757
rs406388
rs1051695
rs599887
rs7611619
rs6873273
rs2130519
rs4072917
rs773125
rs12154492
rs10041403
rs2187388
rs4273233
rs7405987
rs1265565
rs6971441
rs71368508
rs11160618
rs10801274
rs6956399
rs4144686
rs10498754
rs11787216
rs41430945
rs111300896
rs11049380
rs2962853
rs36092039
rs35307180
rs3803286
rs946526
rs2047812
rs33431
rs12808129
rs2155077
rs11246795
rs62502022
rs13246732
rs79966207
rs56094217
rs7728341
rs62124874
rs62317264
rs73482000
rs2170719
rs2467997
rs75231257
rs59768128
rs28590228
rs4777183
rs1203850
rs11736548
rs13060816
rs59259939
rs1466234
rs68018407
rs6440003
rs9881883
rs9878602
rs7335993
rs4529405
rs6992179
rs11125047
rs2332058
rs10145001
rs5343
rs12600322
rs4538234
rs11112945
rs1009368
rs893434
rs13197257
rs9564497
rs12144309
rs7986422
rs9992872
rs10994386
rs10175449
rs2079270
rs10516927
rs2593163
rs12823540
rs698769
rs13214277
rs1342929
rs2243504
rs78706681
rs9905914
rs56391038
rs1623177
rs454568
rs2647258
rs7383874
rs2612607
rs12290967
rs12031155
rs9885441
rs6571932
rs1638526
rs7119426
rs4254636
rs9923598
rs1018466
rs1060402
rs71582886
rs1379511
rs133908
rs6998822
rs858502
rs2301579
rs61094404
rs2192564
rs148736595
rs6593691
rs1494343
rs4567133
rs2545751
rs17767336
rs7133013
rs28412876
rs17669337
rs558706
rs35400008
rs12120481
rs17153017
rs7460852
rs41228
rs1011339
rs76522376
rs778138
rs35764849
rs9935325
rs7468681
rs1712957
rs10840683
rs4778984
rs12139295
rs10065792
rs2360635
rs4902026
rs58768853
rs6656743
rs13226650
rs7196853
rs7555395
rs687094
rs9380526
rs28371453
rs4242551
rs12149498
rs876493
rs4639798
rs13269741
rs16835705
rs4742127
rs7996330
rs62180106
rs2617956
rs1921622
rs12924872
rs7016874
rs10169534
rs6829629
rs17314727
rs11646496
rs3087979
rs9517578
rs13239186
rs11545169
rs12917189
rs9847958
rs717915
rs1366024
rs6704768
rs11204752
rs823114
All
−1.00
−0.75
−0.50
−0.25
0.00
MR leave−one−out sensitivity analysis for
'Self−reported "ability to smell"' on 'Parkinson's Disease'

Supplementary Figure 3 - Forest plot of reverse-causation MR results

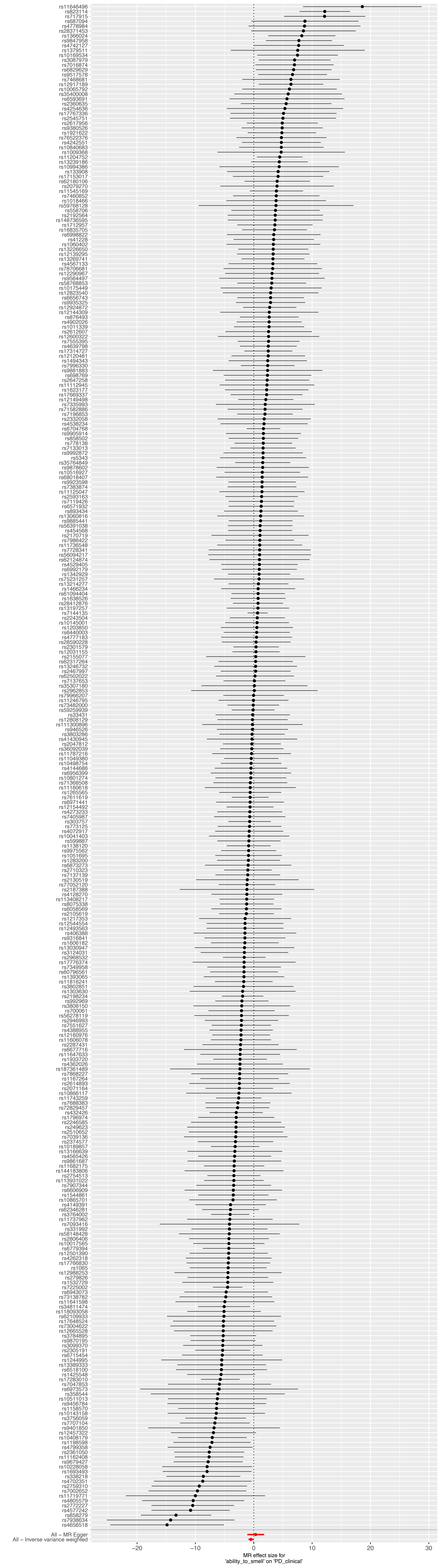
All – MR Egger
All – Inverse variance weighted
−20
−10
0
10
20
30
MR effect size for
'ability_to_smell' on 'PD_clinical'

Supplementary Figure 4 - Scatter plot of reverse-causation MR results

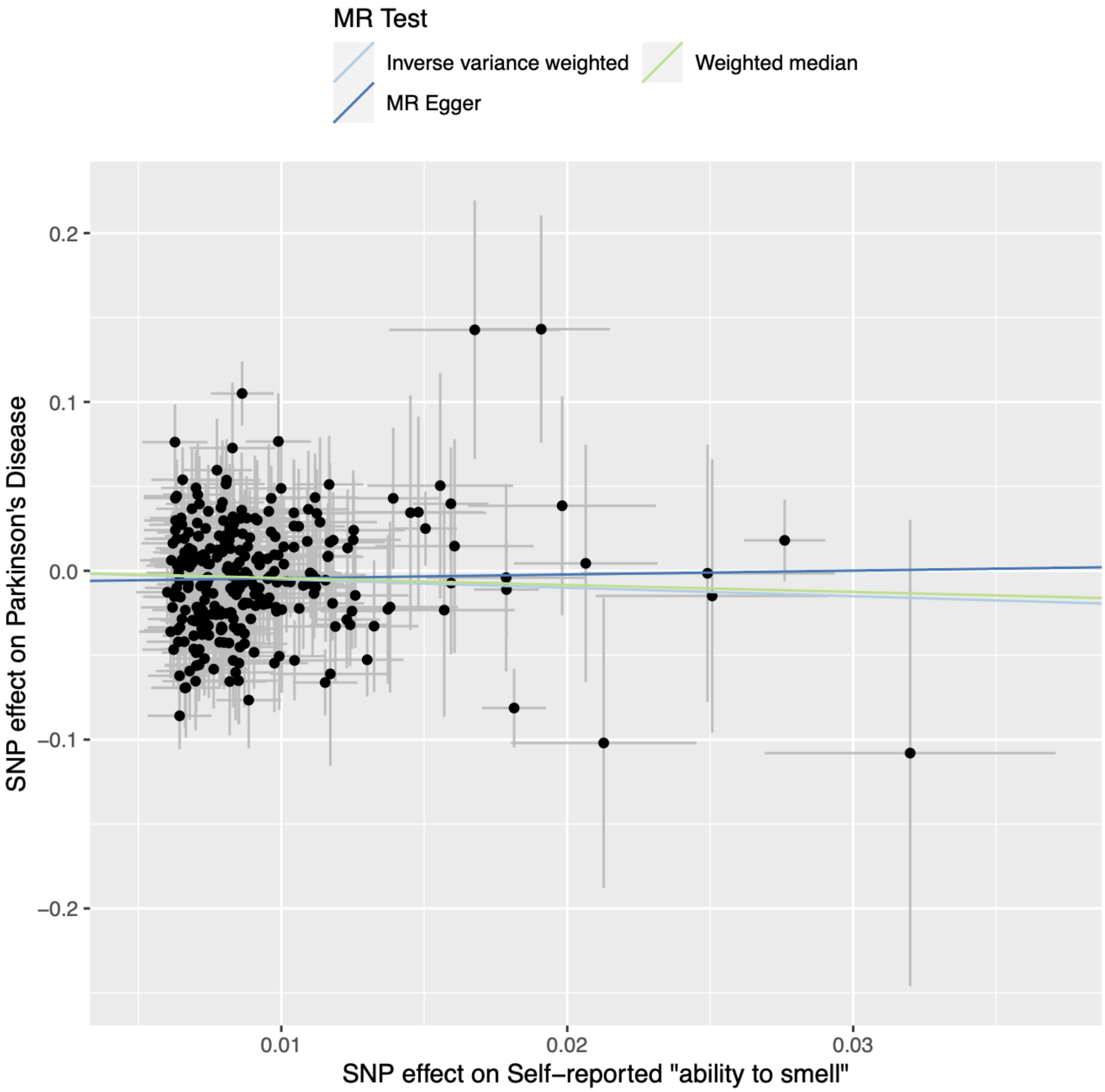

Supplementary Figure 5 - Scatter plot of outlier-removed reverse-causation MR results

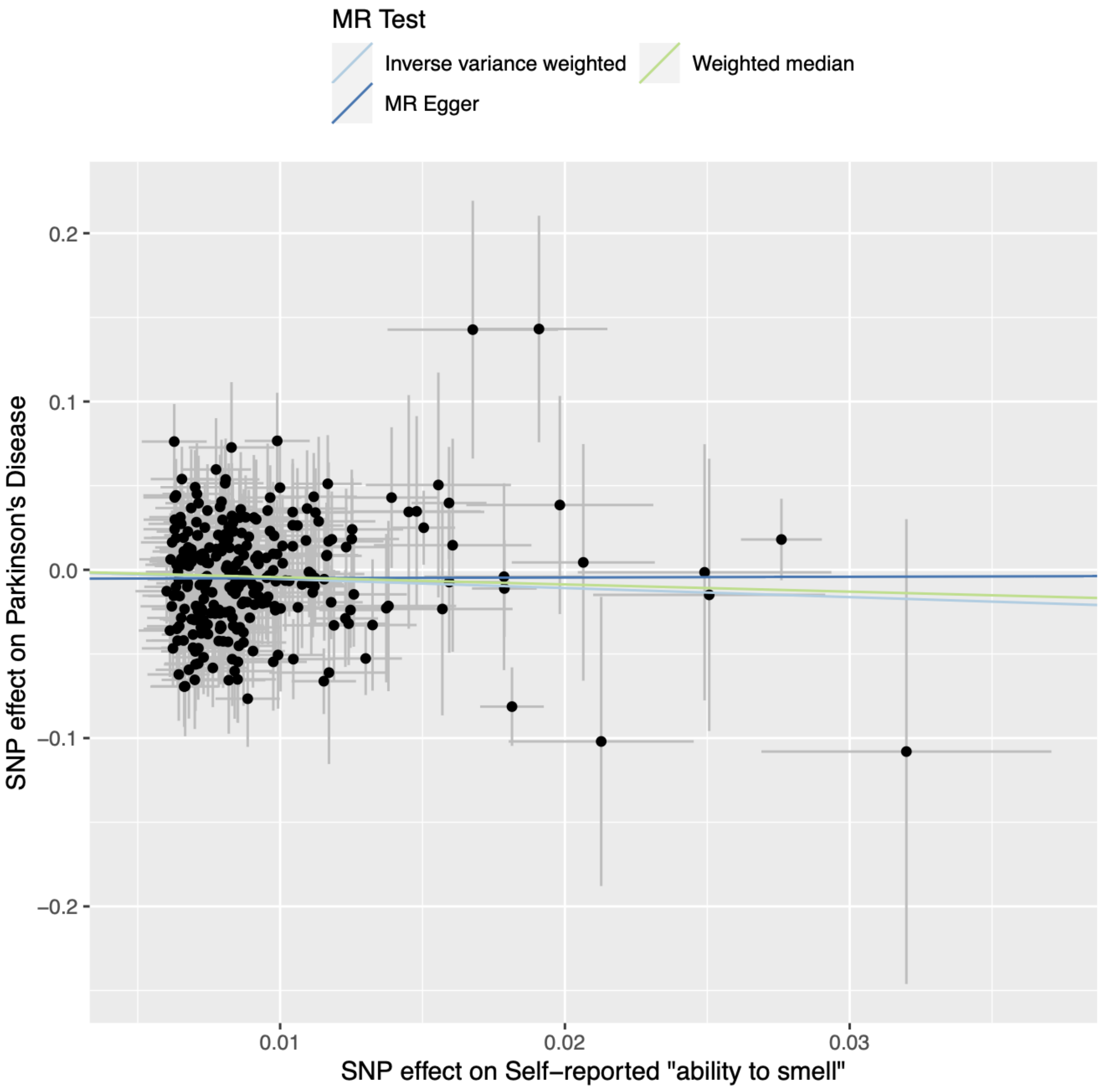

Supplement: Supplement 1 [file media-1.pdf]
